# IgE Immune Complexes Mitigate Eosinophilic Immune Responses through NLRC4 Inflammasome

**DOI:** 10.1155/2023/3224708

**Published:** 2023-10-18

**Authors:** Ece Oylumlu, Goksu Uzel, Lubeyne Durmus, Ceren Ciraci

**Affiliations:** Molecular Biology and Genetics Department, Istanbul Technical University, Istanbul 34469, Turkey

## Abstract

Immune complexes (ICs) skew immune responses toward either a pro- or anti-inflammatory direction based on the type of stimulation. Immunoglobulin E (IgE) is associated with Th2 immune responses and known to activate innate immune cells. However, roles of antigen (Ag)-specific-IgE ICs in regulating human eosinophil responses remain elusive; therefore, this study builts upon the mechanism of which ovalbumin (Ova)-IgE ICs affects eosinophilic responses utilizing human EoL-1 cell line as a model. Eosinophils are granulocytes functioning through pattern recognition receptors (PRRs) and destructive granule contents in allergic inflammation and parasitic infections. One of the PRRs that eosinophils express is NLRC4, a member of the CARD domain containing nucleotide-binding oligomerization (NOD)-like receptor (NLR) family. Upon recognition of its specific ligand flagellin, NLRC4 inflammasome is formed and leads to the release of interleukin-1*β* (IL-1*β*). We exhibited that Ova-IgE ICs induced the NLRC4-inflammasome components, including NLRC4, caspase-1, intracellular IL-1*β*, and secretion of IL-1*β*, as well as the granule contents MMP9, TIMP1, and TIMP2 proteins via TLR2 signaling; these responses were suppressed, when NLRC4 inflammasome got actived in the presence of ICs. Furthermore, Ova-IgE ICs induced mRNA expressions of *MMP9*, *TIMP2*, and *ECP* and protein expressions of MMP9 and TIMP2 in EoL-1 through Fc*ɛ*RII. Interestingly, TLR2 ligand and Ova-IgE ICs costimulation elevated the number of CD63+ cells, a degranulation marker, as compared to the native IgE. Collectively, our findings provide a mechanism for the impacts of Ova-IgE ICs on eosinophilic responses via NLRC4-inflammasome and may help understand eosinophil-associated diseases, including chronic eosinophilic pneumonia, eosinophilic esophagitis, eosinophilic granulomatosis, parasitic infections, allergy, and asthma.

## 1. Introduction

Allergic inflammation is an important pathophysiological condition associated with parasitic infections and certain diseases, including asthma and allergic rhinitis. These pathologic conditions are mainly mediated by immunoglobulin E (IgE)-dependent mechanisms, which influence the functioning of several immune cells, including eosinophils through the interactions with specific receptors [1, 2]. IgE and IgE immune complexes (ICs) (antigen (Ag) + Ag-specific IgE) can bind to two different receptors known as the low-affinity IgE receptors (Fc*ε*RII; CD23) expressed on various antigen-presenting cells (APCs) ranging from B cells to eosinophils and high-affinity IgE receptors (Fc*ε*RI) expressed on mast cells and basophils [3]. Fc*ε*RII is involved in numerous allergic responses such as the transportation of IgE-allergen complexes across the gut and airways in epithelial cells. Fc*ε*RII is also critical in internalization of IgE-allergen complexes and presenting Ags on major histocompatibility complex-II (MHC-II) molecules to the T-helper type 2 (Th2) cells [3]. Although, Fc*ε*RI has no enzymatic activity, its engagement with IgE and IgE ICs induces degranulation and release of histamine and prostaglandin from mast cells and also activation of Th2 responses by mediating interleukin-4 (IL-4), IL-13, and granulocyte macrophage colony-stimulating factor (GM-CSF) secretion by basophils [3, 4]. Recent studies have revealed that the presence of allergen-specific IgE ICs in the airways enhanced the allergic airway inflammation [5], as well as the release of eosinophilic peroxidase through the interaction of eosinophils with different classes of ICs [6].

IgE differs from IgM, IgD, IgG, and IgA by its *ε* heavy chains. Structural studies of IgE-Fc alone, and when it is bound to Fc receptors Fc*ε*RI and Fc*ε*RII/CD23, showed a bent Fc conformation, as well as allosteric communications between the two distant receptor-binding sites [7]. The structure of IgE molecule is distinguished from that of IgG by the additional heavy constant domain and by the lack of a hinge region in the *ε*-chain. Although, IgE-Fc portion has a symmetrical chemical sequence, its three-dimensional structure is asymmetrical [8, 9]. These findings are attributed to the flexibility of IgE and underscore its ability to undergo extreme conformational changes. Furthermore, unbound IgE-Fc maintains a bent configuration, while IgE-Fc is partly bent when it is bound to omalizumab's Fab fragment, which is an anti-IgE in clinical use for the treatment of allergy [10]. Flexibility is certainly crucial for IgE function [11, 12]; however, it could also be favorable for allosteric interference to inhibit IgE activity for therapeutic strategies. These structural features of IgE could be utilized in the formation of IgE-mediated ICs. Therefore, IgE ICs can reprogram immune responses developed by Fc*ε*RI and Fc*ε*RII/CD23 expressing cells such as mast cells, basophils, and eosinophils and can be exploited to tailor the immune responses for the benefit of the host.

Eosinophils are the innate immune cells involved in the initiation and the maintenance of Th2 immune responses [13]. Even though eosinophils only constitute 1%–6% of all white blood cells, they may develop extensive inflammatory responses with their plenty of secretory granules containing eosinophil cationic proteins (ECP), eosinophil-derived neurotoxins (EDN), various cytokines, chemokines, matrix metalloproteinases (MMPs), and tissue inhibitors of metalloproteinases (TIMPs) [13, 14]. Eosinophils secrete these preformed granule contents immediately by degranulation in response to certain stimuli [13]. Eosinophils mediate inflammatory responses through their PRRs, including Toll-like receptors (TLRs) and Nod-like receptors (NLRs) [15]. NLRs are the cytosolic PRRs that are activated by either pathogen-associated molecular patterns (PAMPs) or danger-associated molecular patterns (DAMPs) [16]. The NLR family has 22 members in humans [17]. Several members of the NLR family, including NLRP1, NLRP3, NLRP6, NLRP7, NLRP12, NLRC4, and NAIP, are known to form multimeric protein complexes called “inflammasomes,” which regulate the activation of caspase-1, thereby the production of biologically active interleukin-1*β* (IL-1*β*), IL-18 [18, 19]. The inflammasome formed by NLRC4 responds to type III secretion system (TTSS) rod and needle protein or bacterial flagellin (FLA) [20]. NAIP is required for the recognition of bacterial proteins and NLRC4 inflammasome activation. The NAIP-NLRC4 inflammasomes recognize Gram-negative bacteria such as *Salmonella typhimurium*, *Pseudomonas aeruginosa*, and *Legionella pneumophila* [21, 22]. In one of our recent studies, we reported the involvement of NLRC4 in various eosinophilic functions through the induction of surface molecules, including CD63, CD80, and Fc*ε*RII [23].

We herein show that ovalbumin (Ova)-specific IgE ICs increase the eosinophilic granule contents, including MMP9, TIMP2, and ECP, in TLR2-primed EoL-1 eosinophil cell line after induction of Fc receptors. Also, unlike native IgE, in the absense of a specific Ag in the vicinity, TLR signaling along with Ova-IgE ICs gave rise to EoL-1 cells degranulation. Interestingly, Ova-IgE ICs suppressed the NLRC4 protein and the activation of NLRC4 inflammasome in EoL-1 human eosinophils, which, in turn, resulted in diminished production and secretion of IL-1*β*, MMP9, TIMP1, and TIMP2 responses. Taken together, these data suggest a new role for NLRC4, an alternative pathway for human eosinophilic functions via Ag-specific IgE ICs.

## 2. Materials and Methods

### 2.1. Cell Culture

The EoL-1 cell line was used as model for human eosinophils [24]. The cells were cultured in RPMI 1640 medium (PAN-Biotech GmbH, Aidenbach, Germany) supplemented with 10% inactivated newborn calf serum, 2 mM glutamine, 1 mM sodium pyruvate, 0.1 mM nonessential amino acids, 100 U/ml penicillin, 100 *μ*g/ml streptomycin, and 10 mM HEPES and incubated at 37°C and 5% CO_2_.

### 2.2. Immune Complexes and Cell Stimulations

To form Ova-IgE ICs, chicken egg Ova (Grade V) (Sigma-Aldrich, St. Louis, MO, USA) and mouse anti-Ova IgE monclonal antibody (Chondrex, Inc.) were mixed at a 1 : 10 (*μ*g Ova: *μ*g IgE) ratio and incubated for 30 min at room temperature. Native human IgE was used in the experiments. To activate NLRC4 inflammasome formation, EoL-1 cells were seeded in antibiotic-free medium and stimulated with TLR2 agonist PAM3CSK4 as a first signal (1 *μ*g/ml; InvivoGen, San Diego, CA, USA) and 4 hr poststimulation, TLR2 primed cells were transfected with FLA (100 ng/ml; InvivoGen) using lipofectamine 2000 (Invitrogen) both with and without IgE-Ova immune complexes. Cells were lysed 24 hr after PAM3CSK4 stimulation. Supernatants were collected and assayed for IL-1*β* and IL-10 secretion and gelatinolytic activity.

### 2.3. Immunoblotting

Thirty microgram of proteins per lane were determined using the Pierce BCA Protein Assay Kit (Thermo Fisher Scientific). Proteins were separated on 10%–12% sodium dodecyl sulfate (SDS)-polyacrylamide gels and transferred to polyvinylidene difluoride (PVDF) membrane (Bio-Rad Laboratories, Hercules, CA, USA). For protein detection, the membranes were first probed with primary antibodies against anti-NLRC4 (BioLegend, San Diego, CA, USA), anticaspase-1 (Abcam), anti-IL-1*β* (CST), anti-MMP2 (CST), anti-MMP9 (CST), anti-TIMP1 (CST), and anti-TIMP2 (CST). Anti-GAPDH (CST) and antivinculin (CST) antibodies were used as housekeeping proteins. The membranes were then incubated with horseradish peroxidase (HRP)-conjugated antirabbit and antimouse secondary antibodies (CST). Subsequently, the protein bands were visualized by electrochemiluminescence (ECL) (Roche, Mannheim, Germany) using the ChemiDoc XRS + System (Bio-Rad Laboratories). Band intensities were quantified using Image Lab Software (Bio-Rad Laboratories).

### 2.4. Flow Cytometry

After stimulation, EoL-1 cells were washed in FACS buffer (phosphate-buffered saline (PBS) with 2% fetal bovine serum (FBS)) and incubated for 45 min at 4°C in the dark with the following antibodies: APC antihuman CD63 (BioLegend), APC antihuman CD69 (BioLegend), APC antihuman CD23 (BioLegend), and APC antihuman Fc*ε*R1*α* (BioLegend). Cell staining was assessed by flow cytometry on an Accuri C6 Flow Cytometer (BD Biosciences). FACS analysis was performed with FlowJo software (Tree Star Inc., Ashland, OR, USA).

### 2.5. Cytokine Measurement

The Sandwich ELISA method was used to measure the levels of cytokines secreted from EoL-1 cells. Nunc MaxiSorp 96-well plates were coated with purified antihuman IL1-*β* and IL-10 (BioLegend) antibodies diluted in PBS (1 : 250) and incubated overnight at 4°C. The wells were washed with PBST and blocked with blocking solution (10% FBS containing PBS) for 1 hr at room temperature. Biotin-conjugated antihuman IL-1*β* and IL-10 (BioLegend) antibodies diluted in blocking solution (1 : 250) were added and incubated for 1 hr at room temperature. HRP Avidin D diluted in blocking solution (1 : 2,000) was added and incubated for 30 min at room temperature. The TMB Peroxidase Substrate and TMB Peroxidase Substrate Solution B were mixed in 1 : 1 ratio and added to each well. After the color change was observed, the reaction was stopped by adding 1 N HCl. The absorbance value was measured at 450 nm with the spectrophotometer.

### 2.6. Real-Time RT-PCR

Total RNA was isolated from samples (three wells from 24 well plates, three replicates per each treatment) using RNAqueous© (Ambion, Austin, TX, USA) according to manufacturer's instructions. All RNA samples were DNase treated with DNA-Free (Ambion) according to manufacturer's instructions before quantitative PCR.

MMP9 primers: (F 5′-TTCTCCAGAAGCAACTGTCC-3′, R 5′-TAGGTGATGTTGTGGTGGTG-3′) [25], MMP2 primers: (F 5′-CCGTGTTTGCCATCTGTTTTAG-3′, R 5′-AGGTTCTCTTGCTGTTTACTTTGGA-3′) [26], TIMP1 primers: (F 5′- AATTCCGACCTCGTCATCAG-3′, R 5′-TGCAGTTTTCCAGCAATGAG-3′) [27], TIMP2 primers: (F 5′- TTCATTCGTCTCCCGTCTTT-3′, R 5′- ACCAACGTGTGTGGATCAAA-3′) [28], hNLRC4 set 1 primers (variants 1, 2, 3): (F 5′-GTGTTCTCCCACAAGTTTGA-3′, R 5′-AGTAACCATTCCCCTTGGTC-3′), hNLRC4 set 2 primers (variant 4): (F 5′-AAGATGAATGAAGAAGATGCTATAA-3′, R 5′-ATCAAGAATGCTCAGTTTGACC-3′), proteoglycan 2 (ECP) primers (F 5′-AAACTCCCCTTACTTCTGGCT-3′, R 5′-GCAGCGTCTTAGCACCCAA-3′) [29], eosinophil-derived neurotoxin (EDN) primers (F 5′-AGATCAACGACGAGACCCTC-3′, R 5′-GCTGAAGGGGTATGGAGACT-3′), and hypoxanthine guanine phosphoribosyl transferase (HPRT) 1 primers (F 5′-GACCAGTCAACAGGGGACAT-3′, R 5′-AACACTTCGTGGGGTCCTTTTC-3′).

Each RT-PCR reaction was performed as previously described [30, 31]. The mRNA levels for the target gene corrected to those for the housekeeping gene (HPRT) were calculated by subtracting their corresponding cycle threshold (*Ct*) before and after stimulation using the following formula:

(1) $$\begin{array}{c} \text{Before stimulation},\Delta Ct_{\text{control}}=Ct_{\text{target gene control}}-Ct_{\text{HPRT control}}. \end{array}$$

(2) $$\begin{array}{c} \text{After stimulation},\Delta Ct_{\text{stimulated}}=Ct_{\text{target gene stimulated}}-Ct_{\text{HPRT stimulated}}. \end{array}$$

The fold change in mRNA was determined by: Fold change 2^*Ct*(stimulated)−*Ct*(control)^. Experiments were performed at least twice, and one representative experiment is depicted. Results were expressed as fold change in expression of stimulated cells relative to nonstimulated cells.

### 2.7. Gelatin Zymography

MMP2 and MMP9 activities secreted from EoL-1 cells were investigated by gelatin zymography technique. Cell supernatants were electrophoresed into 7.5% (w/v) polyacrylamide gel copolymerized with gelatin (3 mg/ml) under nonreducing conditions. After electrophoresis, gels were incubated in renaturing buffer (2.5% Triton X-100) two times for 30 min at room temperature. After renaturation, gels were developed in zymogram activation buffer (50 mM Tris-HCl, 0.2 M NaCl, 5 mM CaCl_2_, 1 *μ*M ZnCl_2_) for two to four nights at 37°C. After incubation, gels were stained with Coomassie Brilliant Blue solution for 2 hr and visualized using the ChemiDoc XRS + System (Bio-Rad Laboratories). Band intensities were quantified using Image Lab Software (Bio-Rad Laboratories). Human recombinant MMP9 (hrMMP9) (10 ng; Sigma-Aldrich) was used as a standard control.

### 2.8. Statistical Analyses

Statistical analyses were performed using an unpaired two-tailed *t*-test or two-way ANOVA. ( ^*∗*^*P* ≤ 0.05,  ^*∗∗*^*P* ≤ 0.01, and  ^*∗∗∗*^*P* ≤ 0.001).

## 3. Results

### 3.1. Ova-IgE (ICs) Enhanced NLRC4 Inflammasome Components in TLR2-Primed EoL-1 Cells In Vitro

**Figure 1:**
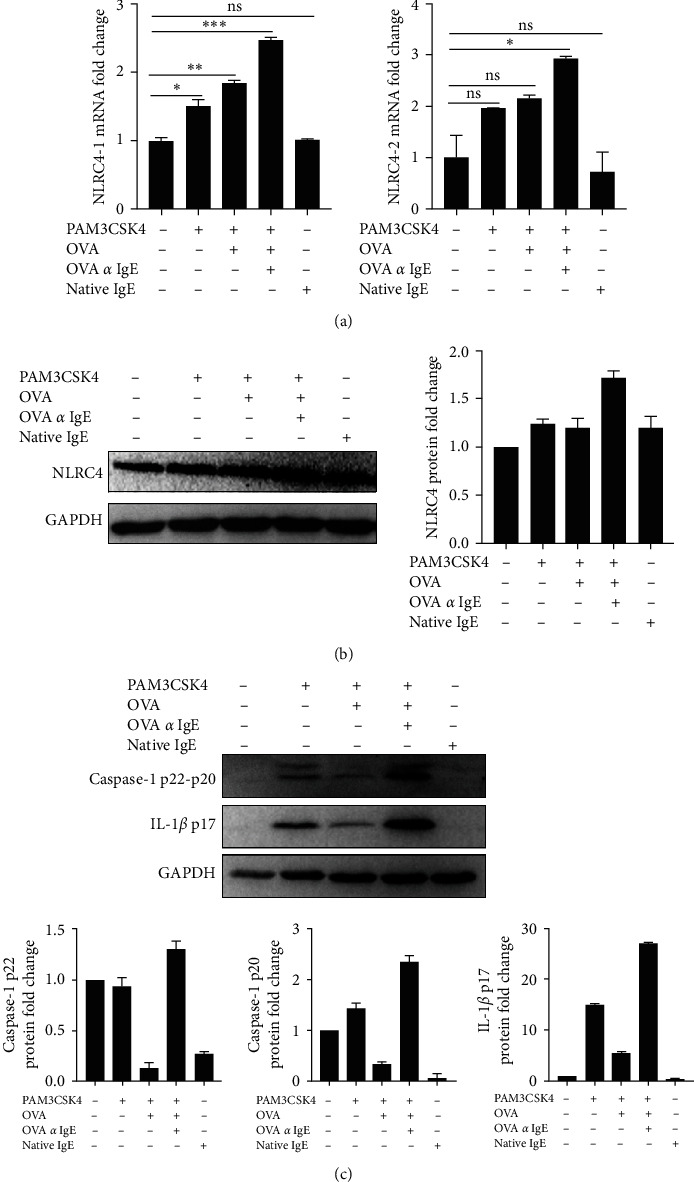
Ova-IgE ICs upregulated NLRC4 inflammasome components in TLR2-primed EoL-1 cells. EoL-1 human eosinophils were primed with TLR2 ligand PAM3CSK4 (1 *µ*g/ml) and treated with IgE (10 *µ*g)-Ova (1 *µ*g) ICs. (a) NLRC4 mRNA expressions by real-time qPCR after Ova-IgE ICs treatment in TLR2-primed EoL-1 human eosinophils. Values represent the mean ± SD and are representative of three separate experiments. Student's *t*-test shows the significant difference between treated and nontreated cells. (b and c) Western blot and corresponding densitometry analyses of cell lysates (30 *µ*g). NLRC4, caspase-1, and IL-1*β* protein expressions were immunoblotted. Values represent the mean ± SD and are representative of three separate experiments.

We used EoL-1 human eosinophilic leukemia cell line as a model for the investigation of eosinophilic functions for four main reasons: (1) their rapid (within hours) responsiveness to broad range of stimuli, (2) their expression profile of PRRs whose roles in regulating eosinophilic responses are largely unknown, (3) their ability to express human eosinophilic pan markers, including Siglec-8 and IL5R [23, 24], and (4) eosinophils' sparsity in blood. Our previous study already exhibited that NLRC4 is expressed and inducible in EoL-1 cells at both the mRNA levels and protein levels [23]. Since both Ag-specific IgE and eosinophils are the participants of the Th2-mediated allergic inflammation, we used Ova and Ova-specific IgE to form Ova-IgE ICs. Former studies demonstrated that IgG ICs-Fc*γ*R engagement during TLR priming steps inhibits the assembly and activation of the NLRP3 inflammasome complexes [32, 33]. From this point of view, we assessed how Ova-IgE ICs affected the innate immune responses in EoL-1 cells through NLR inflammasomes. Utilizing PAM3CSK4, a TLR2 ligand, as the priming signal and intracellular FLA from *S. typhimurium* as the activation signal for NLRC4 inflammasome, we tested eosinophil responses in the presence of Ova-IgE ICs and IgE alone. First, we examined the influence of Ova-IgE ICs on the mRNA and protein expression of NLRC4 in EoL-1 cells after the priming step through TLR2 signaling. Because the human *NLRC4* gene has four transcript variants, two different primer sets were used to detect all *NLRC4* transcripts and showed that Ova-IgE ICs upregulated the expression of NLRC4 as compared to nonstimulated cells and TLR2-primed cells (Figure 1(a)). Moreover, we observed that Ova-IgE ICs increased NLRC4 protein and NLRC4 inflammasome proteins, including caspase-1 and mature IL-1*β* in EoL-1 cells when compared to nonprimed control cells and TLR2-primed cells (Figures 1(b) and 1(c)). Collectively, these findings indicated a role for antigen-specific IgE ICs in regulating NLRC4 inflammasome activation in the absence of NLRC4's ligand FLA.

### 3.2. Ova-IgE ICs Altered Human EoL-1 Eosinophils' Granule Contents, Degranulation, MMP9 Expression, and Secretion In Vitro

**Figure 2:**
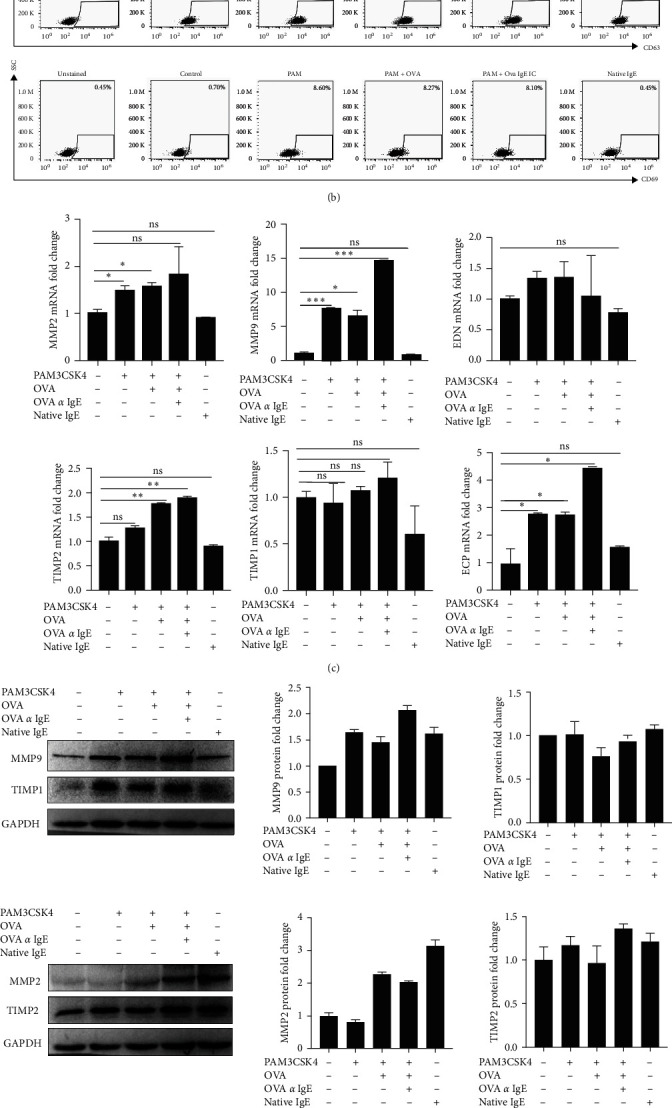
Ova-IgE ICs increased Fc*ε*R2 surface expression and eosinophil granule contents, but did not affect the degranulation. EoL-1 human eosinophils were primed with TLR2 ligand PAM3CSK4 (1 *µ*g/ml) and treated with IgE (10 *µ*g)-Ova (1 *µ*g) ICs. (a) Surface expression of Fc*ε*R1*α* and Fc*ε*R2. (b) CD63 and CD69 on EoL-1 eosinophils were determined by flow cytometry. EoL-1 cells were gated based on their size and granularity using FSC-H/SSC-H by removing debris and doublet cells using FSC-A/FSC-H. Single cells were subgated using Fc*ε*R1*α*, Fc*ε*R2, CD63, and CD69. Percentages within the gates indicate the proportion of these receptor expressions in EoL-1 cell population. Values represent the mean ± SD and are representative of two separate experiments. (c) *MMP2*, *MMP9*, *TIMP1*, *TIMP2*, *EDN*, and *ECP* mRNA expressions by real-time qPCR after Ova-IgE ICs treatment in TLR2-primed EoL-1 human eosinophils. Values represent the mean ± SD and are representative of two separate experiments. Student's *t*-test shows the significant difference between stimulated and nonstimulated cells. (d) Western blot and corresponding densitometry analyses of cell lysates (30 *µ*g). MMP2, MMP9, TIMP1, and TIMP2 protein expressions were immunoblotted. Values represent the mean ± SD and are representative of three separate experiments. (e) Pro- and active MMP9 enzyme activity from the supernatants of the stimulated and nonstimulated cells was measured by gelatin zymography assay. Lane 1: Human recombinant MMP9 (hrMM9) (10 ng) was used as a positive control.

Because NLRC4 inflammasome activation augmented the allergic reactions by inducing low affinity receptor Fc epsilon receptor 2 (Fc*ε*R2, aka CD23) [23] and ICs elevated immune responses via binding to Fc receptors, we texted the effects of Ova-IgE ICs on the expression of high affinity Fc*ε*RI and low affinity IgE receptor Fc*ε*RII in EoL-1 cells. We demonstrated that TLR2 activation along with Ova-IgE ICs treatment significantly upregulated the expression of Fc*ε*R2 in EoL-1 cells while this costimulation did not change the number of Fc*ε*R1-expressing cells (Figure 2(a)).

Eosinophils can be activated by a variety of stimuli, including allergens, infections, tissue injury, and tumors. This activation leads eosinophils to release their granule contents with degranulation and secrete variety of cytokines, chemokines, and enzymes. CD63 is the member of transmembrane-4 glycoprotein superfamily and has been depicted as a potential surface marker for eosinophil degranulation, and CD69 is a type II transmembrane protein and expressed on eosinophils as an early activation marker [34, 35]. Thus, we sought to address how Ova-IgE ICs altered the activation and degranulation in EoL-1 cells by analyzing the expression of CD63 and CD69, respectively, after TLR2 activation and Ova-IgE ICs treatment using flow cytometry (Figure 2(b)). Our data clearly exhibited that costimulation with TLR2 ligand and Ova-IgE ICs increased the expression of CD63+ cells as compared to nontreated cells; however, ICs per se did not have any impact on the CD63 expression.

Similarly, we examined the eosinophil activation by CD69 surface marker expression and determined a higher number of CD69-expressing cells than the cells cotreated with TLR2 ligand and Ova-IgE ICs. Surprisingly, native IgE, when there is no Ova Ag in the vicinity, induced the number of CD63+ cells, but not CD69 in a manner that does not require an inflammatory pathway.

We further tested the regulatory roles of Ova-IgE ICs in the context of eosinophil granule proteins, including MMPs and TIMPs. MMPs are calcium-dependent zinc-containing endopeptidases consisting of at least 24 members in vertebrates involved in extracellular matrix (ECM) degradation [36, 37]. Numerous studies have shown that MMPs, particularly MMP9 and MMP2, are involved in the release or activation of cytokines, chemokines, and growth factors that have roles in innate and adaptive immunity, inflammation, bone and airway remodeling, angiogenesis cancer progression, invasion, and metastasis [38–40]. Conceivably, MMP9 has also been shown to contribute to eosinophilic asthma-related airway remodeling and inflammation [41, 42]. In addition to the current literature, one of our recent studies also suggested that NLRC4 inflammasome promotes MMP9 expression and secretion but not MMP2 in EoL-1 human eosinophils [23]. TIMPs, on the other hand, are known for their regulatory function in the ECM metabolism, tissue remodeling, cellular behavior, and, even more interestingly, in the defense mechanisms [43, 44]. For these reasons, we first investigated the effects of Ova-IgE ICs on the mRNA expression of *MMP9*, *MMP2*, TIMP1, and *TIMP2*. Quantitative analysis determined that *MMP9* and *TIMP2* mRNA expressions were significantly upregulated after Ova-IgE ICs treatment in EoL-1 cells through TLR2 induction, while the mRNA expression of *MMP2* and *TIMP1* did not significantly change (Figure 2(c)). Consistent with the mRNA expression, MMP9 protein expression was also upregulated following the Ova-IgE ICs treatment in EoL-1 cells (Figure 2(d)). Next, we performed zymogram assay to measure the gelatinase activity of MMP9 and MMP2 in response to Ova-IgE ICs and results from this particular experiment exhibited that Ova-IgE ICs did not significantly affect the MMP2 in the TLR2-activated cells (data not shown); however, proteolytic activity of MMP9 increased when compared to nontreated cells (Figure 2(e)). Additionally, we evaluated the impact of Ova-IgE ICs on TIMP1 and TIMP2 proteins on EoL-1 cells, which pointed out that although MMP9, TIMP1 and TIMP2 were all coexpressed in EoL-1 cells, unlike MMPs, TIMPs' protein expressions did not significantly change (Figure 2(d)). Last, we measured the mRNA expression levels of *ECP* and *EDN*, which are also important granule proteins of eosinophils (Figure 2(c)). Interestingly, unlike *EDN*, *ECP* expression levels were significantly upregulated by Ova-IgE ICs in EoL-1 cells, suggesting that ICs exclusively regulated the discharge of eosinophil granule contents depending upon the context of stimuli (Figure 2).

### 3.3. Ova-IgE ICs Suppressed NLRC4 Inflammasome-Mediated Inflammation in EoL-1 Cells

**Figure 3:**
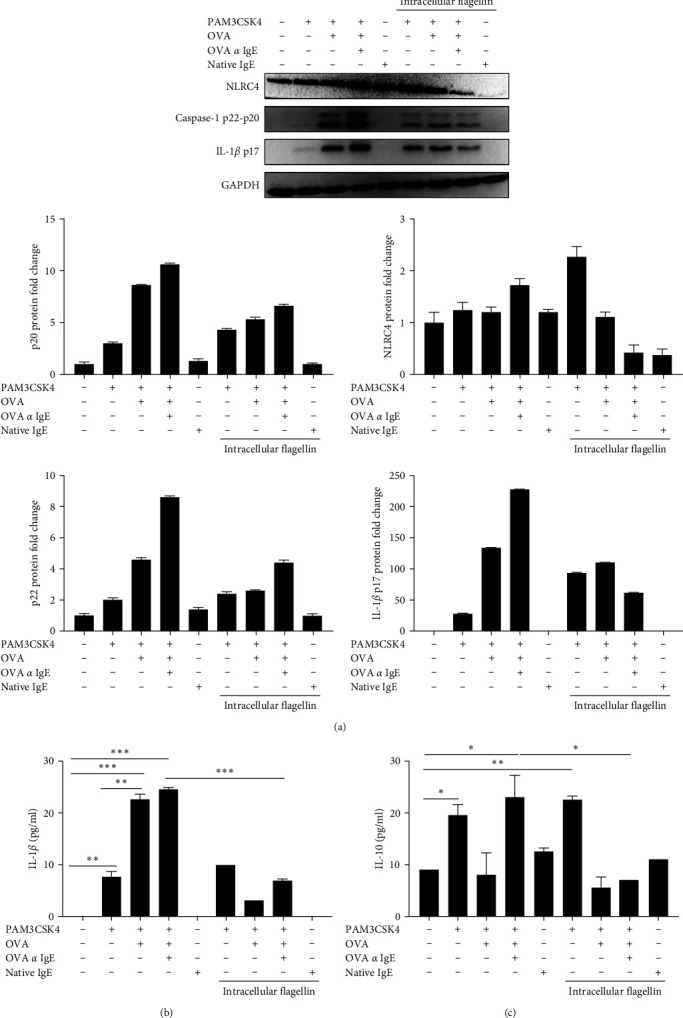
Ova-IgE ICs suppress NLRC4 inflammasome-mediated inflammation in EoL-1 cells. EoL-1 cells were treated with IgE (10 *µ*g)-Ova (1 *µ*g) immune complexes. For NLRC4 inflammasome induction, TLR2-primed EoL-1 cells were transfected with 100 ng/ml FLA and incubated for 20hr. (a) Western blot and corresponding densitometry analyses of cell lysates (30 *µ*g). NLRC4, caspase-1, and IL-1*β* protein expressions were immunoblotted. Values represent the mean ± SD and are representative of three separate experiments. (b) Supernatants were collected and analyzed for IL-1*β* and (c) IL-10 by ELISA. Experiments were carried out in duplicate. Values represent the mean ± SD and are representative of two separate experiments.  ^*∗∗*^*P* ≤ 0.01 by Student's *t*-test.  ^*∗*^*P* < 0.05,  ^*∗∗*^*P* < 0.01, and  ^*∗∗∗*^*P* < 0.001.

As dichotomous effects of ICs on inflammatory responses through inflammasome activation were formerly reported [32, 33] and most importantly, a functional role was attributed to NLRC4 inflammasome in eosinophils [23], we reasoned that Ova-IgE ICs might modulate the eosinophilic inflammatory responses in a NLRC4-dependent manner. Hence, we transfected EoL-1 cells with FLA to induce NLRC4 inflammasome activation in TLR2-primed EoL-1 cells and examined the effects of Ova-IgE ICs after intracellular FLA stimulation by transfection. Expectedly, transfection of TLR2-primed EoL-1 cells with FLA resulted in the secretion of IL-1*β*; however, the presence of Ova-IgE ICs significantly inhibited NLRC4 inflammasome proteins (Figure 3(a)), which, in turn, gave rise to diminished IL-1*β* maturation and secretion from EoL-1 cells (Figure 3(b)). We previously demonstrated that the suppression of lipopolysaccharide (LPS)-induced IL-12 production by IgG-ICs entailed the increased IL-10 production [33]; thus, we addressed whether Ova-IgE ICs inhibit NLRC4 inflammasome-mediated immune responses by elevating IL-10 production in EoL-1 human eosinophils. Of note, IL-1*β* and IL-10 have rather low expression profiles in eosinophils as compared to other innate immune cells such as dendritic cells and macrophages [45]. Concomitantly, IL-1*β* and IL-10 had relatively low expression profiles in EoL-1 eosinophils, yet both cytokines were still in our detection range. We detected that IL-10 production increased in the presence of Ova-IgE ICs via TLR2 signaling pathway; however, it was suppressed when NLRC4 inflammasome was activated by FLA (Figure 3(c)). Interestingly, native IgE and FLA costimulation, in the absence of the specific Ag Ova, did not interfere with IL-10 production while it completely suppressed IL-1*β* secretion; a result attributing an important role to NLRC4 in the regulation by IgEs (Figure 3).

### 3.4. Ova-IgE ICs Reduced Intracellular MMP9, TIMP1, and TIMP2 Proteins via NLRC4 Inflammasome

**Figure 4:**
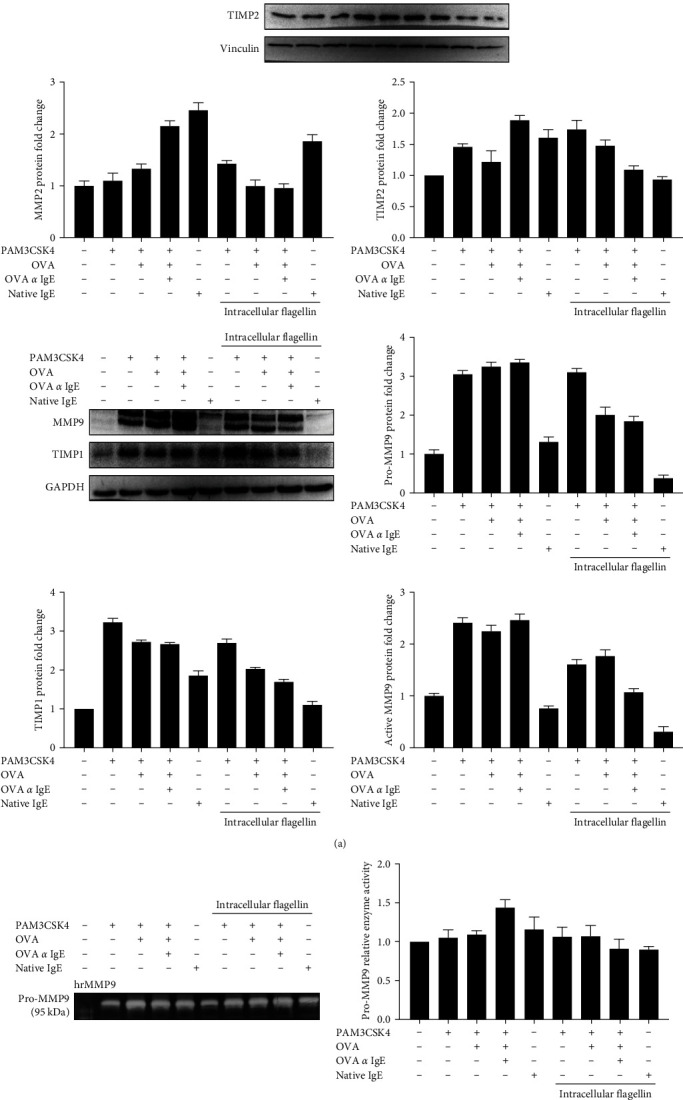
Ova-IgE ICs reduced MMP2, MMP9, TIMP1, and TIMP2 proteins but not MMP9 secretion and activation. EoL-1 cells were treated with IgE (10 *µ*g)-Ova (1 *µ*g) immune complexes. For NLRC4 inflammasome induction, TLR2-primed EoL-1 cells were transfected with 100 ng/ml FLA and incubated for 20 hr. Western blot and corresponding densitometry analyses of cell lysates (30 *µ*g). (a) MMP2, MMP9, TIMP1, and TIMP2 protein expressions were immunoblotted. Values represent the mean ± SD and are representative of three separate experiments. (b) Pro- and active MMP9 enzyme activities from the supernatants of the stimulated and nonstimulated cells were measured by gelatin zymography assay.

Since we already revealed that NLRC4 inflammasome activation upregulated the MMP9 expression, secretion, and proteolytic activity in EoL-1 cells [23] and herein showed that Ova-IgE ICs induced MMP9 at both the mRNA and protein levels in TLR2-primed EoL-1 cells, we further examined the effects of Ova-IgE ICs on NLRC4 inflammasome-mediated MMPs and TIMPs expressions. Interestingly, Ova-IgE ICs reduced the expression of MMP2, MMP9, TIMP1, and TIMP2 proteins when NLRC4 inflammasome induced in EoL-1 cells (Figure 4(a)); moreover, the proteolytic acitivity of MMP9 slighly reduced, but no MMP2 activity was detected (data not shown) after Ova-IgE ICs and NLRC4 ligand treatment in EoL-1 cells (Figure 4(b)). Taken together, these results indicate a role for Ag-specific IgE ICs in regulating eosinophilic functions through a mechanism that involves NLRC4 inflammasome.

## 4. Discussion

Eosinophils are essential in the development of Th2 immune responses associated with parasitic infections and allergic pathologies [13]. As innate immune cells, eosinophils possess numerous TLRs and NLRs, which recognize PAMPs or DAMPs resulting in the generation of immune responses against parasites and allergens. In addition to their intracellular organelles, eosinophils contain secretory granules, which store preformed cytokines, chemokines, and immunomodulatory molecules such as MMPs [13]. Although eosinophils constitute a very low number of leukocytes, they have a potential to compensate for their scarcity in blood by their ability to activate extensive physiological and biological processes with the release of their preformed granule contents by degranulation in response to certain stimuli [13]. Because of eosinophils' low number in leukocytes, limited life expectancy in vitro, as well as the low transfection efficiency [46], EoL-1 cell line was employed to investigate the eosinophilic functions in the present study. We previously reported that EoL-1 cells displayed eosinophilic characteristics, including the expression of IL-5R*α* and Fc receptors [23]. The aim of the current study was to better understand the bidirectional mechanisms of Ova-IgE ICs on innate immune responses in human eosinophil-like cells to be able to extend these findings to human diseases. Our results indicated that Ova-IgE ICs elevated NLRC4 mRNA and protein expressions in EoL-1 cells without a necessity of the second signal for the caspase-1 cleavage, as well as IL-1*β* maturation.

IgE-dependent mechanisms and circulating ICs are associated with both initiation and progression of allergic and autoimmune diseases through the engagement with Fc receptors. Due to the increasing evidence on the airway inflammation as a result of allergen-specific IgE ICs [5] and elevated eosinophil numbers in bronchoalveolar lavage (BAL) fluid in sensitized mice with IgE ICs, we examined the cross talk between IgE receptors and inflammation. Our results suggested an induction of Fc*ε*RII expression on TLR2-primed EoL-1 cells after Ova-IgE ICs treatment, while Fc*ε*RI expression did not change.

Overall, our study sought to understand the mechanism of action in which the ICs reprogram responses developed by eosinophil-like cells. The vast majority of eosinophils reside in the gastrointestinal tract [47], whereas a low number of cells circulate in the peripheral blood making these cells challenging to work with; hence, as mentioned above, we used EoL-1 cells as they endogenously express the molecules in the scope of this study. Clinical outcomes of Ova-IgE ICs should clearly be tested in mouse models of asthma, allergy, or airway hyperresponsiveness. However, it could be speculated that IgE-formed ICs hold great potential for the treatment of not only asthma and allergy but also all eosinophil-associated diseases (EADs), including cancer in the context of host's inflammation.

Translation to clinical studies requires meticulous monitoring of patients and analyzing the immunological reactions of type I hypersensitivity following the administration of IgE ICs due to the likelihood of degranulation in basophils or mast cells as well as eosinophils. Functional tests may monitor predisposition to induce basophil activation and/or mast cell degranulation in blood and sera. These measurements could be performed at different time points of IgE ICs administration. Monitoring of patients would include signs of type I hypersensitivity, changes in serum levels of proteases, antigen-specific IgE, and autoantibodies to the specific antigen. Particularly, serum proteases, including *β*-tryptase, can be a marker for degranulation during clinical studies referring to reactions from type I hypersensitivity [48, 49].

Since, elucidating regulatory roles of IgE ICs for the treatment of allergy, asthma, and eosinophil-related diseases requires animal testing first, only clinical data for the treatment of asthma would be for the anti-IgE antibodies. One example is that omalizumab which is a clinically tested and approved humanized IgG1 that binds to circulating IgE, and unlike any other anti-IgEs, it does not bind to IgE that is already bound to Fc*ε*RI on the surface of cells [50], thereby decreases the cell-bound IgE and tissue infiltration of eosinophils and mediator release, resulting in the relief of allergic inflammation and asthma symptoms. Even for the Food and Drug Administration (FDA) approved omalizumab, adverse effects such as anaphylaxis, a severe systemic allergic reaction, has been reported [51].

This study depicts that EoL-1 cells not only express NLRC4, but also NLRC4 inflammasomes can be activated upon treatment with TLR2 ligand PAM3CSK4 and intracellular FLA resulting in the cleavage of caspase-1 and IL-1*β* [23]. Ova-IgE ICs along with NLRC4's ligand (intracellular FLA) diminished the intracellular caspase-1 and IL-1*β* at protein levels, which was also confirmed by significant reduction in IL-1*β* secretion. Earlier studies suggested that the costimulation of ICs with TLR ligands induced IL-10 expression in macrophages, as well as Ag-specific IgG ICs alone induced IL-10 production [33, 52]. In line with the literature, our results indicated that Ova-IgE ICs increased IL-10 secretion from TLR2-primed EoL-1 cells, whereas NLRC4 inflammasome activation led to a significant reduction in IL-10 secretion from EoL-1 cells. Surprisingly, native IgE and NLRC4 inflammasome activation did not affect the IL-10 production, while IL-1*β* and caspase-1 were significantly suppressed, a result referring an important role to NLRC4 in the regulation by IgEs.

Because CD63 and CD69 are commonly used as a degranulation and eosinophil activation markers, respectively [35, 53], we assessed the discharge of granule content by CD63 expression and human EoL-1 eosinophil activation by CD69 upon TLR2 and Ova-IgE ICs costimulation, which increased the number of CD63- and CD69-expressing cells as compared to nontreated cells; however, Ova-IgE ICs per se did not affect their expressions. Interestingly, native IgE, when there is no Ova antigen in the vicinity, significantly elevated the number of CD63+ expressing cells, but not CD69, in a manner independent of inflammatory pathways.

MMP2 and MMP9 (gelatinases) are the most studied and well-characterized MMPs whose expressions and activations have been addressed in neoplastic pathologies and inflammatory disorders, including asthma [37]. Several studies elucidated the regulations of MMP9 through TLR2 activation in human monocytes [54]. The relationship between TLR signaling and MMP9 expression in immune cells has been established; however, we indicated a mechanism for ICs that involve NLRC4 inflammasome complexes, which reduced the expression of MMP2, MMP9, TIMP1, and TIMP2 proteins upon activation in TLR2-primed Ova-IgE ICs-treated EoL-1 cells.

Additionally, IgE ICs can either enhance or suppress the eosinophilic immune responses depending on the timing and the type of infection in the host. IgE-allergen ICs are known to induce a rapid activation of basophils and mast cells. Moreover, APCs can present innocuous antigens to T cells by the help of IgE. These findings underlined the importance of proximity of IgE epitopes on the antigen, which greatly affects the subsequent shape of antigen and antibody ICs, as well as the potency of antigen, which then determines the severity of allergic reactions [55]. Therefore, it is reasonable to speculate that epitope positioning of IgE binding to the antigen could be engineered to change the antigenic potency of allergens and could be utilized for the design of allergy vaccines. In addition to the epitope positioning, glycosylation profile of IgE molecules is of great importance for its structure and function in clinical applications. IgE is highly glycosylated and carbohydrates can affect its affinity for the antigen, bioavailability for the tissues, and pharmacokinetics. For example, high mannose structure at Asn394 has been proposed to have significance in its function [56, 57].

IgE immunobiology in the context of treatment strategies yet to be discovered. Applications of IgE-mediated ICs or IgE per se in clinical settings are not only limited to the allergy or asthma diseases but rapidly extending to the other diseases, including the treatment of cancer. In this regard, AllergoOncology is emerging as a new field, which suggests the exploitation of IgE antibodies to treat malignant diseases due to the presence of modulatory mechanisms for IgE antibodies [58]. In this study, we exhibited the modulatory mechanisms for IgE when it is in complex with an innocuous antigen with respect to eosinophilic responses to inflammatory stimuli. Further studies will most definitely be needed to determine the means by which IgE ICs can remodulate immune responses to maximize the benefit for the host.

## 5. Conclusions

In conclusion, our study suggested a novel role for NLRC4 inflammasome in the suppression of eosinophil responses by antigen-specific IgE ICs. It is noteworthy that the clinical trials and drug development studies on EAD are limited and nonspecific [59]; this study raises the potential of new inflammatory targets for the treatment of eosinophil-related diseases stemming from deficient eosinophil responses and expands the knowledge for future studies to dissect the mechanism further.

## Data Availability

The data used to support the findings of this study are included within the article.
